# Fatal adverse events of rivaroxaban combined with aspirin: an analysis using data from VigiBase

**DOI:** 10.1007/s00228-022-03357-4

**Published:** 2022-07-01

**Authors:** Qingxia Zhang, Qian Ding, Suying Yan, Qun-Ying Yue

**Affiliations:** 1 Department of Pharmacy, National Clinical Research Center for Geriatric Disease, Xuanwu Hospital Capital Medical University, Beijing, China; 2 School of Pharmaceutical Science, Capital Medical University, Beijing, China; 3 Uppsala Monitoring Centre, Uppsala, Sweden

**Keywords:** Rivaroxaban, Aspirin, Fatal, Adverse drug events, Medication errors, VigiBase

## Abstract

**Purpose:**

The aim of this study was to analyze the clinical characteristics of fatal adverse events (AEs) of rivaroxaban combined with aspirin and to underline the importance of the rational use of drugs.

**Methods:**

The WHO global database of reported potential side effects of medicinal products (VigiBase) was searched for fatal AEs in the combined use of rivaroxaban and aspirin, and the clinical characteristics of those cases with sufficient information (vigiGrade completeness score ≥ 0.80) were analyzed.

**Results:**

By January 19, 2020, 2309 fatal adverse event reports of rivaroxaban combined with aspirin from 21 countries were entered in VigiBase. One hundred and twenty cases contained further information, of which 42 were female (35%) and 78 were male (65%). The median age was 75 (range 34 to 93) years, and 109 cases (91%) were elderly patients (≥ 65 years). The AEs listed in the fatal case reports included bleeding in 114 cases (mainly intracranial hemorrhage and gastrointestinal hemorrhage, 59 and 46 respectively, accounting for 88%) and ischemic events in six cases (ischemic stroke in three, acute myocardial infarction in two, myocardial infarction combined with acute liver failure in one). Among the patients with bleeding events, 108 (95%) had existing risk factors for bleeding or for interacting with aspirin or rivaroxaban. These may be divided into the following: diseases (hypertension, renal impairment, history of stroke, peptic ulcer, or previous bleeding), drugs (high dose aspirin, antiplatelet drugs, anticoagulants, P-gp inhibitors/CYP3A4 inhibitors, non-steroidal anti-inflammatory drugs, steroids, and selective serotonin reuptake inhibitors), or other factors (e.g., elderly, low body weight, or excessive intake of ginger, fish oil, or alcohol). There were 45 cases with two or more of these risk factors in addition to rivaroxaban and aspirin. Patients with ischemic events are often in very high-risk groups of atherosclerotic cardiovascular disease (ASCVD) or self-discontinuation of treated drugs. Medication errors occurred in 24 patients (20%): excessive treatment in 17 cases, contraindication in three, frequency error in two, excessive treatment combined with contraindication in one, and self-discontinuation in one.

**Conclusions:**

Fatal AEs related to rivaroxaban combined with aspirin, including bleeding and ischemic events, have been reported mostly in the elderly, and sometimes involved medication errors. The fatal AEs mainly manifested as serious bleeding, and most of them occurred in patients with concurrent multiple risk factors. Monitoring coagulation during rivaroxaban treatment is recommended in very high-risk ASCVD populations, and attention should be paid to prevention of medication errors.

## Introduction 

Atrial fibrillation (AF) and coronary heart disease (CHD) share many risk factors, so these two diseases often coexist. The incidence of AF in patients with CHD is 6 to 21% [1], while the incidence of CHD in patients with AF is 20 to 30% [2–4]. Direct oral anticoagulants (DOACs) are the first choice for stroke prevention in patients with non-valvular atrial fibrillation (NVAF), especially for the elderly [5, 6]. Rivaroxaban is one of the commonly used DOACs. Aspirin is a widely used antiplatelet drug for CHD [7]. Therefore, rivaroxaban and aspirin are often taken concomitantly in NVAF combined with percutaneous coronary intervention (PCI), or with stable CHD accompanying high ischemic risk without high bleeding risk [8]. Previous studies have shown that aspirin is a separate factor which affects rivaroxaban bleeding [9], and aspirin combined with rivaroxaban may not bring net clinical benefits for stable CHD [10]. In order to understand the potential risks for death in patients treated with rivaroxaban and aspirin, we undertook this study to analyze the clinical characteristics of these fatal cases from the WHO global database of reported potential side effects of medicinal products (VigiBase).

## Methods

### Data source

The data for this study were derived from a search of VigiBase on 19 January 2020 using active ingredients acetylsalicylic acid (aspirin) and rivaroxaban reported as suspected/interacting. All reports with a fatal outcome were extracted.

### Data extraction

Adverse events (AEs) were classified using the System Organ Classification (SOC) and Preferred Term (PT) of the international Medical Dictionary for Regulatory Activities (MedDRA) [11]. All the useful variables contained in the reports were considered, including the following: (1) case information: the country from which the report came and the category of the reporter; (2) patient information: age, gender, body weight, height, relevant medical history, and alcohol intake; (3) medication information: indications for suspected and concomitant medication, dosage, and medication timing; (4) description of AEs: clinical manifestations, the tests and procedures performed, treatment, and outcome. The criterion for well-documented cases [12] was vigiGrade completeness score ≥ 0.80 (allowing analysis of the clinical characteristics).

### Risk factor identification

The diseases, drugs, and other risk factors that lead to bleeding/thrombosis were determined according to the drug instructions [13, 14] of rivaroxaban and aspirin, relevant guidelines [5, 15, 16], and literature [17–21], combined with the HAS-BLED score [22]. The risk factors of well-documented cases were analyzed.

## Results

From 1968 to 19 January 2020, VigiBase had received 134,916 reports of potential side effects of rivaroxaban, of which 14,178 with a fatal outcome; 20,462 of rivaroxaban combined with aspirin, of which 2309 had a fatal outcome. The 2309 fatal cases were reported from 21 countries, including 968 male (59%), 1323 female (40%) patients, and in 18 (0.8%) the information on sex was not provided. The median age was 77 (range 9 to 97), and majority of the patients (1936, 84%) were 65 years or over.

Of the 2309 reports, 120 (5.2%) were classed as well-documented, from 12 countries. The patients comprised 78 males (58%) and 42 females (42%). The median age was 75 (range 34 to 93) years, and majority (109, 91%) were 65 years or over. The indications of rivaroxaban were AF in 99, pulmonary embolism (PE)/deep venous thrombosis (DVT) in nine, arrhythmia in two, and one each for the following: acute coronary syndrome (ACS), coronary artery stenosis, arterial thrombosis, and unknown. The indications of aspirin were cerebral infarction in eight; AF in six; CHD and ST-Elevation/Non–ST-Elevation Myocardial Infarction in four each; ACS, cardiac disorder, PCI, and peripheral obliterative arteriopathy in three each; ischemic heart disease in two; angina pectoris, arteriosclerosis, stable coronaropathy, pericarditis, and PE in one each; and unknown in 79.

The AEs reported in these fatal cases were bleeding in 114 cases. There were 59 with intracranial bleeding (combined with gastrointestinal bleeding in two, hematuria in one, and intraventricular bleeding in one), and 46 with gastrointestinal bleeding (combined with renal injury/failure in five, hematuria in two, intracranial bleeding in two, liver failure with renal failure in one, urinary sepsis in one, internal bleeding in one, and secondary to myocardial infarction in one). In addition, there were single cases of abdominal bleeding, pleural bleeding, retroperitoneal hematoma, alveolar hemorrhage, hematoma after falling, hematuria, epistaxis, hemorrhagic arteriovenous malformation, internal bleeding, myocardial infarction secondary to internal bleeding, and hemorrhagic shock. One hundred and eight cases (108/114, 94.7%) had diseases (hypertension in 21; stroke in seven; abnormal renal function and hemorrhage in two each; peptic ulcer in one), drugs (antiplatelet drugs in 16: clopidogrel in 11, larger dosage of aspirin in 5; anticoagulants in 3: low molecular weight heparin (LMWH), heparin, and warfarin in 1 each; non-steroidal anti-inflammatory drugs (NSAIDs) in 4: metamizole, meloxicam, indomethacin, and celecoxib in 1 each; P-glycoprotein (P-gp) inhibitor/Cytochrome P450 3A4(CYP3A4) inhibitor in 6: dronedarone in 4, amiodarone in 2; steroids in 1: prednisone; selective serotonin reuptake inhibitors(SSRIs) in 1: citalopram), or other risk factors (age ≥ 65 years old in 103; low body weight ≤ 50 kg in 5; alcoholism in 1; ginger in 1; fish oil in 1) that increased the risk of bleeding. In addition to rivaroxaban and aspirin, 45 patients were compromised by two or more bleeding risk factors, some up to six at the same time.

The AEs in the remaining six fatal cases were ischemic events: three cases of ischemic stroke, two of acute myocardial infarction, and one of myocardial infarction complicated with acute liver failure. Most of them (5/6) were very high-risk patients with atherosclerotic cardiovascular disease, except one ischemic stroke patient after self-discontinuation of rivaroxaban. The other two elderly patients with ischemic stroke had a history of ischemic stroke, which was the high-risk population for stroke. Three patients with acute myocardial infarction had a history of myocardial infarction events.

Medication errors occurred in 24 cases (20%). There were 17 cases of excessive treatment (seven of AF complicated with cerebral infarction and five of AF using anticoagulation combined with antiplatelet drugs, two of a long course of dual antiplatelet after PCI, one case of AF complicated with ischemic cardiomyopathy, one case of CHD, and one case of AF complicated with stable CHD). Three cases included a contraindication (combined with dronedarone), and two had frequency errors (rivaroxaban was given 10 mg/15 mg twice a day for stroke prevention in patients with AF). There was one case of excessive treatment plus a drug contraindication (combined with dronedarone, AF patients using anticoagulation and antiplatelet), and finally one case of insufficient treatment (self-discontinuation).

## Discussion

Fatal AEs related to aspirin combined with rivaroxaban included bleeding and ischemic events, most of which were bleedings. Most of the bleeding events were associated with multiple risk factors that increase bleeding. In addition to the factors listed in the HAS-BLED score [22] (hypertension [uncontrolled, > 160 mmHg systolic, abnormal renal and liver function, stroke, bleeding history or predisposition, labile INRs], elderly [> 65 years], drugs [antiplatelet agents, nonsteroidal anti-inflammatory drugs], or alcohol concomitantly), combination with the following drugs also affects coagulation: steroids (prednisone), SSRIs (citalopram), and drugs that inhibit the metabolism of rivaroxaban (dronedarone and amiodarone) [5]. Some patients take two antiplatelet drugs or one antiplatelet drug with NSAIDs at the same time, but the HAS-BLED score is the same as that of one antiplatelet drug or NSAIDs alone. Therefore, it cannot reflect the cumulative effect of multiple drugs. Adverse reactions of systemic steroids therapy (such as prednisone) include a tendency to bleed [18]. SSRIs may affect platelet function and carry the risk of bleeding [17]. Amiodarone is a moderate P-glycoprotein (P-gp) inhibitor. It has mild drug interaction with rivaroxaban, which may increase the bleeding risk [5]. It is recommended to use the HAS-BLED score to assess the risk of bleeding [5]; however, for patients with risk factors not considered in the score, individualized administration should be considered [23]. Therefore, ORBIT scores was recommended in the UK [24], which however is still controversial [25].

In addition to the risk factors discussed above, there are other risk factors for bleeding events, such as low body weight [5] and intake of ginger [19, 20] or fish oil [21]. The ROCKET-AF trial [26] demonstrated that rivaroxaban showed similar efficacy and safety in patients weighing less than or equal to 70 kg and greater than 70 kg. The studies by Malik et al. [27] and Lee et al. [28] found that the safety of rivaroxaban in low body weight patients (< 50 kg) was reassuring, but the study had limitations. De Caterina and Lip [29] suggested that the trough concentration level of rivaroxaban can be measured when rivaroxaban is used in low weight people. Arioli et al. [23] believed that DOACs plasma level measurement may become an important tool for making reasonable decisions in emergency situations, such as the management of patients with severe bleeding. The interaction between warfarin and food is well-known and a widespread concern, but interaction between food and DOACs is often ignored. Gresenberger et al. [20] reported a case of hemoptysis caused by a large intake of ginger during treatment of DVT with rivaroxaban, where the platelet function of the patient was still abnormal 3 days after stopping the ginger. Rivaroxaban product information (Lexicomp) [21] mentioned in the interaction section: lipid emulsion (fish oil base) can enhance the effectiveness of anticoagulants, with the risk rating of C grade, which should be monitored during treatment. Most bleeding events occur in elderly patients, in which multiple diseases often coexist, and multiple drugs are used. Patients taking several medicines will be more likely to have adverse bleeding events during rivaroxaban therapy [30]. The European Heart Rhythm Association’s practical guidelines (2021) on the use of non-vitamin K antagonist oral anticoagulants in patients with AF (the 2021 ESC practical guidelines) [5] recommended care during multiple drug treatment or in the presence of two or more bleeding risk factors. Therefore, more attention should be paid to patients with multiple bleeding risks, especially the elderly.

Patients with ischemic events are often in very high-risk groups of ASCVD or self-discontinuation. Compared to warfarin, DOACs have a better safety profile and a more predictable anticoagulant effect, and routine coagulation monitoring is not needed [31]. However, for the very high-risk population of ASCVD, the risk of insufficient anticoagulation is higher. The recommendations of the International Council for Standardization in Haematology (ICSH) on laboratory measurement of DOACs [32] stated that laboratory tests for the evaluation of drug exposure and anticoagulant effect may help clinicians in decision-making in emergency situations, such as acute stroke and excess weight. Self-discontinuation of drugs will lead to failure of anticoagulant treatment. Patient education may improve medication compliance (see ISMP high warning drug patient education for details) [19].

In addition, medication errors are important trigger factors. The proportion of over-treatment in this study is relatively high. For patients of AF or AF complicated with ischemic stroke/stable CHD, the guidelines recommend only anticoagulant therapy [5]. For those without high risk of ischemia after PCI, the time of anticoagulation combined with anti-platelet treatment should be as short as possible, preferably 1 year [8]. The 2021 ESC practical guidelines [5] suggest that the combination of dronedarone should be avoided when using rivaroxaban for anticoagulant treatment, since dronedarone is an inhibitor of P-glycoprotein and CYP3A4, which can increase the plasma concentration of rivaroxaban. When rivaroxaban is used for stroke prevention in AF patients, the 2021 ESC practical guidelines and drug instructions recommend once a day [5, 14].

Improved ischemic risk stratification is recommended to better identify high-risk treatment candidates while avoiding unnecessary treatment in low-risk populations [33]. It is necessary to regularly review the risk factors of bleeding and thrombosis. The 2021 ESC practical guidelines [5] suggest adjusting patient medication lists and the dosage of rivaroxaban (every 4 months in patients who are 75 years and over or frail, and patients with renal insufficiency every “CrCl/10 = minimum review interval (in months)”) to avoid under- or excessive treatment and potential drug/food interactions.

There are some limitations to this study. Well-documented cases were available in just 5% of the reports, 90% of which came from the USA. The degree of underreporting of all suspected adverse reactions to rivaroxaban cases is unknown and likely to be large. There is no comparator in this study, and accurate comparisons between drugs based on spontaneous reports are anyway not possible. Spontaneous reporting systems can, however, detect the new, rare, and serious AEs [34], which are difficult to detect in the clinical trials and practice.

## Conclusion

Fatal AEs related to rivaroxaban combined with aspirin, including bleeding and ischemic events, have been reported mostly in the frail and elderly, and involving medication errors. The fatal AEs mainly manifested as serious bleeding, and most of them occurred in patients with simultaneous multiple risk factors. Monitoring coagulation during rivaroxaban treatment is recommended in patients with multiple bleeding risk factors or very high-risk ASCVD populations, and attention should be paid to prevention of medication errors to ensure patient safety.

## Data Availability

The original contributions presented in the study are included in the article/Supplementary Material. Further inquiries can be directed to the corresponding authors.
